# An Autophagy-Related Long Noncoding RNA Signature Contributes to Poor Prognosis in Colorectal Cancer

**DOI:** 10.1155/2020/4728947

**Published:** 2020-10-21

**Authors:** Jingsun Wei, Xiaoxu Ge, Yang Tang, Yucheng Qian, Wei Lu, Kai Jiang, Yimin Fang, Maxwell Hwang, Dongliang Fu, Qian Xiao, Kefeng Ding

**Affiliations:** Department of Colorectal Surgery and Oncology, Key Laboratory of Cancer Prevention and Intervention, Ministry of Education, The Second Affiliated Hospital, Zhejiang University School of Medicine, Hangzhou, Zhejiang, China

## Abstract

**Purpose:**

Colorectal cancer is one of the most common malignant primary tumors, prone to metastasis, and associated with a poor prognosis. As autophagy is closely related to the development and treatment of colorectal cancer, we investigated the potential prognostic value of long noncoding RNA (lncRNA) associated with autophagy in colorectal cancer.

**Methods:**

In this study, we acquired information on the expression of lncRNAs in colorectal cancer from the Cancer Genome Atlas (TCGA) database and found that 860 lncRNAs were associated with autophagy-related genes. Subsequently, univariate Cox regression analysis was used to investigate 32 autophagy-related lncRNAs linked to colon cancer prognosis. Subsequently, eight of the 32 autophagy-related lncRNAs (i.e., long intergenic nonprotein coding RNA 1503 [LINC01503], ZEB1 antisense RNA 1 [ZEB1-AS1], AC087481.3, AC008760.1, AC073896.3, AL138756.1, AL022323.1, and TNFRSF10A-AS1) were selected through multivariate Cox regression analysis. Based on these autophagy-related lncRNAs, a risk signature was constructed, and the patients were divided into high- and low-risk groups.

**Results:**

The high-risk group's overall survival time was significantly shorter than that of the low-risk group (*p* < 0.0001). Receiver operating characteristic curve analysis was performed to further confirm the validity of the model (area under the curve: 0.689). Moreover, multivariate regression suggested that the risk score was a significant prognostic risk factor in colorectal cancer. Gene set enrichment analysis showed that these gene sets are significantly enriched in cancer-related pathways, such as Kirsten rat sarcoma viral oncogene homolog (KRAS) signaling.

**Conclusion:**

The risk signature of eight autophagy-related lncRNAs has prognostic potential for colorectal cancer. These autophagy-related lncRNAs may play a vital role in the biology of colorectal cancer.

## 1. Introduction

Colorectal cancer, a disease caused by the interaction of genetic and environmental factors, is one of the most common malignancies worldwide [1]. Statistics show that, in 2017, nearly 140,000 individuals in the United States of America were diagnosed with colon cancer, resulting in 50,000 deaths [2]. In China, colorectal cancer is one of the most common tumors, and its incidence is gradually increasing [3]. Surgery combined with postoperative chemotherapy is the current main treatment for colorectal cancer [4]. The lack of early definitive diagnosis and metastasis is the major cause of death due to colon cancer. In 2014, the 5-year survival rate of patients with local disease was 90.3%, while that of patients with local and distant metastases was 70.4 and 12.5%, respectively [5]. Therefore, screening of the colorectal cancer-related risk signature to evaluate the prognosis is of great significance.

Autophagy is a “self-eating” phenomenon in cells, a physiological process in which membranes enclose organelles and proteins in cells and degrade them [6]. The effect of autophagy on cells is dual; it can promote and inhibit the formation and development of tumors and play varied roles in different tumors. In the early stage of tumor development, autophagy plays a role in inhibiting tumor growth. In the later stages, autophagy helps tumor cells grow in a low vascularization environment [7, 8]. In recent years, many studies found targets in autophagy-related pathways to treat cancer [9]. Besides, studies have confirmed that autophagy-related protein Beclin1 is upregulated in colorectal cancer [10]. This finding indicated that autophagy plays a vital role in this disease.

Long noncoding RNA (LncRNA) has a variety of regulatory modes. It mainly interacts with proteins, DNA, or RNA to regulate gene expression at the epigenetic, transcriptional, and posttranscriptional levels, and participates in many biological activities, such as transcriptional activation, transcriptional interference, and nuclear transport [11]. LncRNA can regulate the function and activity of autophagy-related DNA, RNA, or protein or affect autophagy-related stress factors and energy receptors, thereby participating in the regulation of cell autophagy [12, 13]. For example, HAGLROS is a 699 bp lncRNA that can inhibit cell autophagy through the mammalian target of the rapamycin (mTOR) signaling pathway, thereby promoting tumorigenesis and progression [14, 15]. Previous studies have shown that lncRNA can be used as a potential biomarker for COAD prognosis [16]. However, the autophagy-related lncRNAs are rarely studied, the precise mechanisms of autophagy-related lncRNA in colorectal cancer remain unknown; hence, further investigations are warranted.

At present, research on the relationship between autophagy-related lncRNA and tumors has received extensive attention. In this study, autophagy-related lncRNAs may have latent value in the prognosis of patients with colorectal cancer, and they can be potential therapeutic targets. The present study's objective was to establish autophagy-related lncRNA markers and provide clues for understanding their role in colorectal cancer.

## 2. Methods

### 2.1. Acquiring Information of Patients with Colorectal Cancer

The RNA sequence transcriptome data and relevant clinical information of patients with colon adenocarcinoma (COAD) and rectum adenocarcinoma (READ) were acquired from the Cancer Genome Atlas (TCGA; https://cancergenome.nih.gov/) database.

### 2.2. Selection of lncRNA and Autophagy Genes

The TCGA database was used to obtain the profiles of lncRNAs in patients with colorectal cancer. The autophagy genes were obtained from the Human Autophagy Database (http://autophagy.lu/clustering/index.html). All expression data were normalized using the limma package (version 3.22.7). Pearson's correlation test was performed to study the relationship between the autophagy-related genes (ATGs) and lncRNAs. A correlation coefficient  |R2| >0.4 and *p* < 0.001 confirmed a correlation of the lncRNA with an ATG.

### 2.3. Establishment of the Risk Signature

We evaluated the prognostic value of autophagy-related lncRNAs based on the univariate and multivariate Cox regression analyses. We initially identified lncRNAs with *p* < 0.05 through univariate analysis and subsequently incorporated those lncRNAs into a multivariate Cox regression analysis to establish risk scores. The following formula was used to calculate the risk score for each patient:

(1) $$\begin{array}{c} \text{risk score}=\beta_{\text{gene}1}\times \;\exp\;{\text{r}}_{\text{gene}1}+\beta_{\text{gene}2}\times \;\exp\;{\text{r}}_{\text{gene}2}+\ldots +\beta_{\mathrm{gene}n}\times \;\exp\;{\text{r}}_{\text{gene}n}. \end{array}$$

The risk model was constructed to predict the survival of patients with colon cancer through Cox analysis. The risk scores were assigned by linear combination of the expression of the lncRNAs, which was weighted according to the regression coefficient (*β*). The Akaike information criterion was utilized to optimize the risk model. The subgroups (low- and high-risk groups) were established based on the median risk score. The expression level of lncRNA is defined as exp r_gene*n*_.

### 2.4. Gene Set Enrichment Analysis (GSEA)

The gene expression data were explored by GSEA. The GSEA software was downloaded to analyze the gene sets and deduce the function of the data. This approach can be utilized to study whether there was a statistically significant difference between the high- and low-risk groups. In the present study, we verified whether differentially expressed genes between the two subgroups were enriched during the autophagy process.

### 2.5. Cell Culture and Real-Time PCR

All the cells we used were cultured in RPMI‐1640 (Gibco, USA) with 10% fetal bovine serum (FBS, Life Technologies, Carlsbad, CA), 100°U/mL penicillin, and 100 mg/mL streptomycin. The cells were cultured at 37°C with 5% CO_2_. All cells were acquired from the American Type Culture Collection (ATCC, Rockville, MD).

TRIzol Reagent (Invitrogen, USA) was performed to extract the RNA of the cell lines. Then, the cDNAs were acquired by the reverse transcription (PrimeScript™ RT Master Mix, Takara, Japan). Subsequently, qPCR was used using SYBR® Premix Ex Taq™ GC (Takara, Japan). And, the GAPDH was chosen as the internal control. The primers of the eight autophagy lncRNAs are listed in Table S1. The method of ΔCt was applied to analyze the data.

### 2.6. Statistical Analysis

The autophagy-lncRNA co-expression network was constructed using the CYTOSCAPE software (version 3.5.1; https://cytoscape.org/) [17]. Pearson's correlation, Cox regression, and Kaplan-Meier curve analyses were performed using the *R* software (version 3.6; https://www.r-project.org/). The functional annotation indicated that the risk model was effe of the two subgroups was accomplished using the GSEA software (version 4.0.3; http://www.broadinstitute.org/gsea/index.jsp). *p* values < 0.05 denoted statistically significant differences.

## 3. Results

### 3.1. Establishment of the Autophagy lncRNAs Co-Expression Network

A total of 257 ATGs were acquired from the Human Autophagy Database. Subsequently, two expression matrices of lncRNAs and ATGs in colorectal cancer were obtained through the TCGA database. We obtained co-expression results by performing a correlation analysis of lncRNAs and ATGs. The expression network of autophagy lncRNAs was established to discern the lncRNAs associated with autophagy. As a result, 860 autophagy-related lncRNAs were identified based on the filtering criteria of a correlation coefficient <0.4 and *p* < 0.001. According to the value of the correlation coefficient, the top 10 lncRNAs are presented in Table 1.

### 3.2. Construction of a Risk Model Based on Autophagy-Related lncRNAs in Patients with Colorectal Cancer

After constructing the co-expression network, according to the 860 autophagy-related lncRNAs, we further assessed the prognostic value of lncRNAs through univariate Cox regression analysis. A total of 548 patients with colorectal cancer in the TCGA database were used for the analysis. The significance filtering criterion was set at *p* < 0.05, and 32 autophagy-related lncRNAs were identified (Table 2).

Subsequently, multivariate Cox regression analysis was performed to ulteriorly identify the independent prognostic factors among the 32 lncRNAs. When the Akaike information criterion value reached the optimal value, eight of the 32 lncRNAs were identified as independent prognostic factors: long intergenic nonprotein coding RNA 1503 (LINC01503), ZEB1 antisense RNA 1 (ZEB1-AS1), AC087481.3, AC008760.1, AC073896.3, AL138756.1, AL022323.1, and TNFRSF10A-AS1 (Table 3).

**Figure 1:**
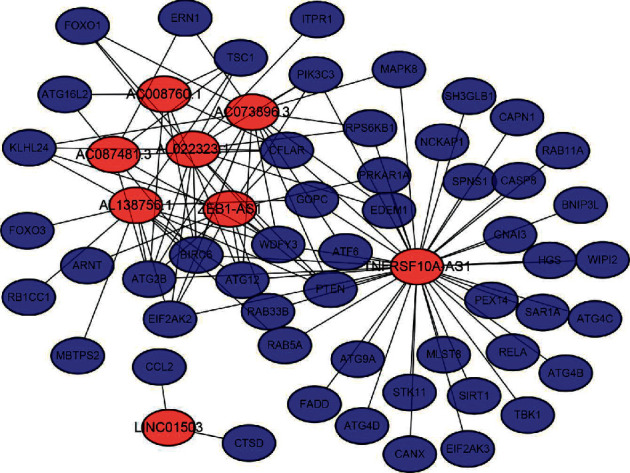
Co-expression network of autophagy genes and related prognostic lncRNAs. Red nodes represent autophagy-related lncRNAs. Blue nodes represent autophagy genes. LncRNA, long noncoding RNA.

**Figure 2:**
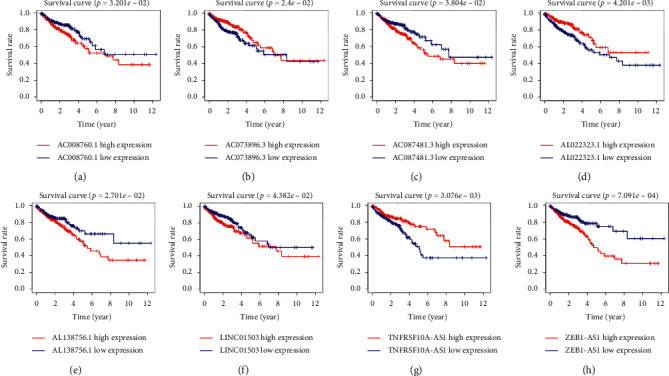
Kaplan–Meier survival curve of the eight selected autophagy-related lncRNAs in colorectal cancer in TCGA database. The Kaplan–Meier survival analysis indicated that patients with high expression of AC008760.1, AC087481.3, AL138756.1, LINC01503, and ZEB1-AS1 had worse prognosis. In contrast, those with high expression of AC073896.3, AL022323.1, and TNFRSF10A-AS1 had better prognosis. LncRNA, long noncoding RNA; TCGA, the Cancer Genome Atlas; LINC01503, long intergenic nonprotein coding RNA 1503; AS1, antisense RNA 1.

**Figure 3:**
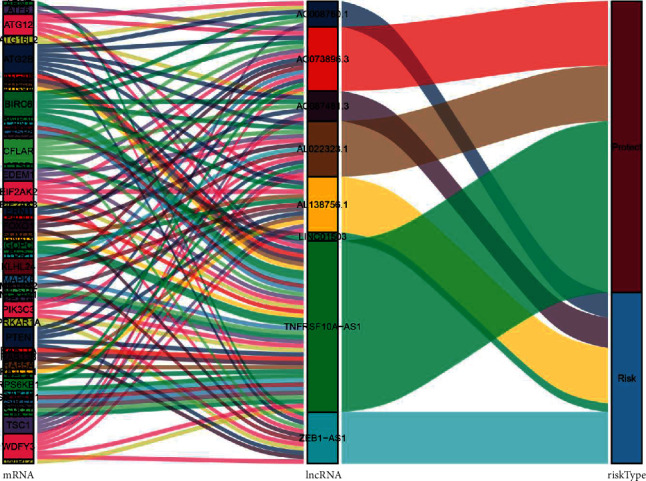
The relationships among autophagy-related genes, autophagy-related lncRNAs, and risk types in the Sankey diagram. lncRNA, long noncoding RNA.

Furthermore, we constructed a network using these eight lncRNAs to exhibit the relationship between the prognostic lncRNAs and ATGs (Figure 1). Next, we verified the relationship between these eight autophagy-related lncRNAs and patients' prognosis with colorectal cancer through a survival curve (Figure 2). All eight lncRNAs were significantly associated with the prognosis of colorectal cancer (*p* < 0.05). According to the risk score acquired from the multivariate Cox regression analysis, we produced a Sankey diagram to exhibit the association among ATGs, autophagy-related lncRNAs, and risk types of lncRNAs (Figure 3). The results showed that three autophagy-related lncRNAs were protective factors (AC073896.3, AL022323.1, and TNFRSF10A-AS1), while the remaining five autophagy-related lncRNAs were risk factors (LINC01503, ZEB1-AS1, AC087481.3, AC008760.1, and AL138756.1).

### 3.3. Prognostic Effects of the Autophagy-Related lncRNA Risk Model on Colorectal Cancer

**Figure 4:**
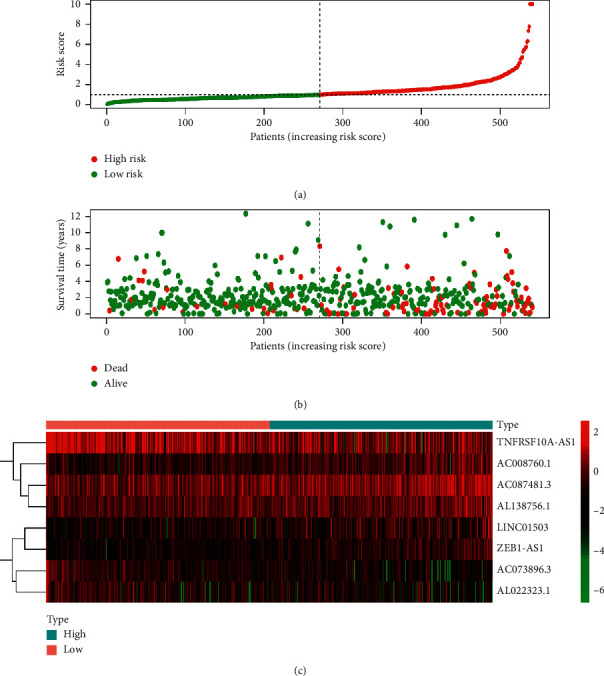
Risk analysis of autophagy-related lncRNAs in patients with colorectal cancer in TCGA database. (a) The risk score in the high- and low-risk groups for the risk signature in colorectal cancer. (b) Survival time of patients with colorectal cancer in high- and low-risk groups. (c) Expression of the eight selected autophagy-related lncRNAs in colorectal cancer. Red color represents increased expression. Blue color represents decreased expression. LncRNA, long noncoding RNA; TCGA, the Cancer Genome Atlas.

**Figure 5:**
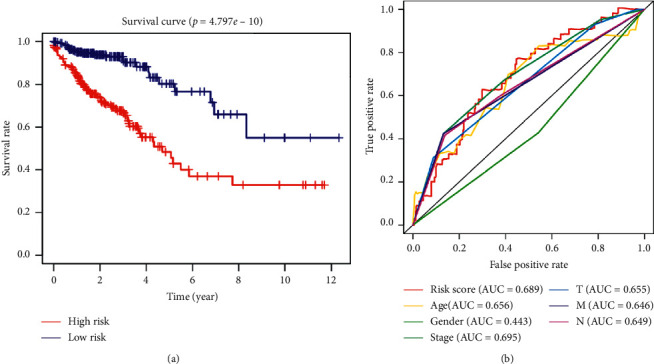
Prognostic value of the risk signature based on coefficients and HR. (a) Survival analysis of the high- and low-risk groups according to the risk model in TCGA database. (b) The accuracy of the model was verified using multi-indicator ROC analysis. HR, hazard ratio; TCGA, the Cancer Genome Atlas; ROC, receiver operating characteristic.

**Figure 6:**
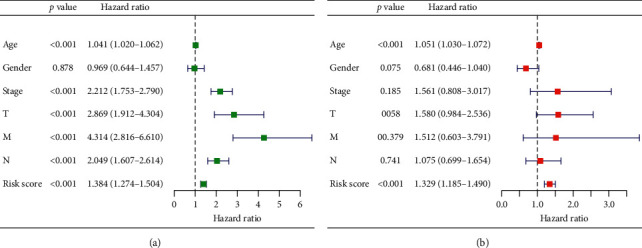
Analysis of the risk signature as an independent prognostic indicator through (a) univariate and (b) multivariate cox regression analyses.

Subsequently, the autophagy-related lncRNA risk model was established based on the risk score. The patients with colorectal cancer were divided into two groups, namely, high- and low-risk groups (Figures 4(a)–4(c)). Consequently, the risk score was a significant predictor of overall survival (OS) in patients with colorectal cancer, with the low-risk group exhibiting a longer OS than that observed in the high-risk group (*p* < 0.05). In addition, the log-rank test was used to obtain the Kaplan–Meier survival curve. The results also suggested that the high-risk group had a worse prognosis (Figure 5(a)). The receiver operating curve was used to measure the risk model's effectiveness, and the area under the curve (0.689) indicated that the risk model was effective (Figure 5(b)). We also used the previously published lncRNAs signature [16] compared with our signature. The results showed that the previously published lncRNA signature was significantly related to OS (Figure S1A); however, the area under the curve was 0.436 (Figure S1B), indicating our signature's superiority based on autophagy-related lncRNAs. In addition, univariate and multivariate Cox regression analyses were performed based on the risk score. Both regression analyses demonstrated that the risk score was a significant prognostic risk factor (univariate regression: *p* < 0.05, hazard ratio: 1.384; multivariate regression: *p* < 0.05, hazard ratio: 1.329) (Figures 6(a) and 6(b)). Collectively, the results suggested that the risk score obtained from our signature can be used as an independent prognostic factor to estimate the OS of patients with colorectal cancer.

### 3.4. The Risk Model Was Closely Related to Clinicopathological Features in Colorectal Cancer

After detecting the significant association between the risk score and the prognosis of colorectal cancer, we further investigated the relationship between the risk score and clinical traits in the clinical information TCGA database. The clinical characteristics included age, sex, stage status, and tumor-node-metastasis status. As shown in Table 4, there was a strong relationship between the risk model of the eight lncRNAs and clinicopathological features, such as stage status and tumor-node-metastasis stage (*p* < 0.05). The risk score in the late stages (stage III-IV) tends to be higher compared with that obtained for the early stages (stage I–II), and it was the same for the *T* (*T*1–2, *T*3–4), *N* (*N*0, *N*1–2), and *M* (*M*0, *M*1) stages. These results suggested that the risk score may be closely related to the progression of colorectal cancer. Interestingly, we also found that males had a higher risk score compared with females; this observation is consistent with the epidemiology of colorectal cancer.

### 3.5. GSEA

**Figure 7:**
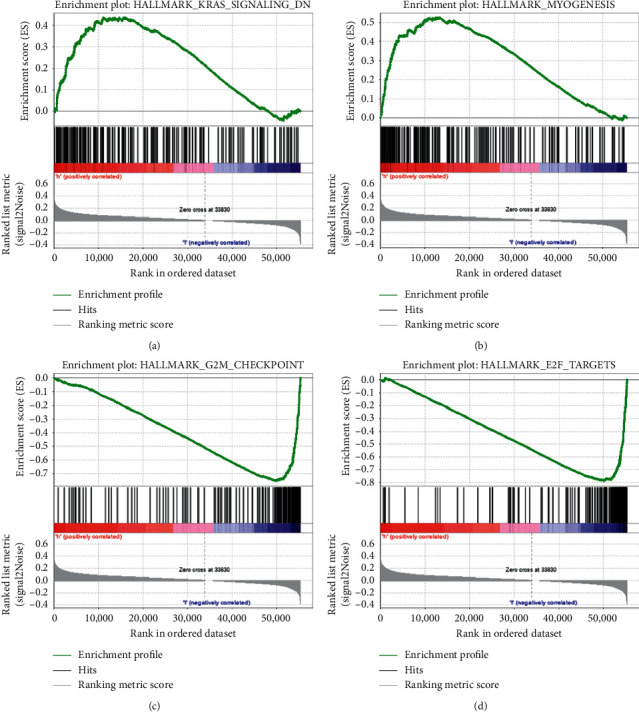
Gene set enrichment analysis showing the enriched hallmark pathways in the two groups based on TCGA database. (a), (b) KRAS and myogenic gene sets were significantly enriched in the high-risk group. (c), (d) G2°M checkpoint and E2F targets gene sets were significantly enriched in the low-risk group. TCGA, the Cancer Genome Atlas; KRAS, Kirsten rat sarcoma viral oncogene homolog.

The GSEA was used to explore further functional annotation. The results showed that the differentially expressed genes were significantly enriched in some tumor-related pathways between the high- and low-risk groups. Twelve gene sets were upregulated in the high-risk group and two gene sets were significantly enriched at nominal *p* value <0.05 and false discovery rate <25%.  Figure 7 shows that Kirsten rat sarcoma viral oncogene homolog (KRAS) signaling and myogenic gene sets were significantly enriched in the high-risk group (Figures 7(a) and 7(b)). The G2°M checkpoint and E2F target gene sets were significantly enriched in the low-risk group (Figures 7(c) and 7(d)). The results based on the autophagy-related lncRNAs showed that the KRAS signaling pathway might play an important role in autophagy in colorectal cancer.

### 3.6. Expression of the lncRNAs in Cell Lines of Colorectal Cancer

**Figure 8:**
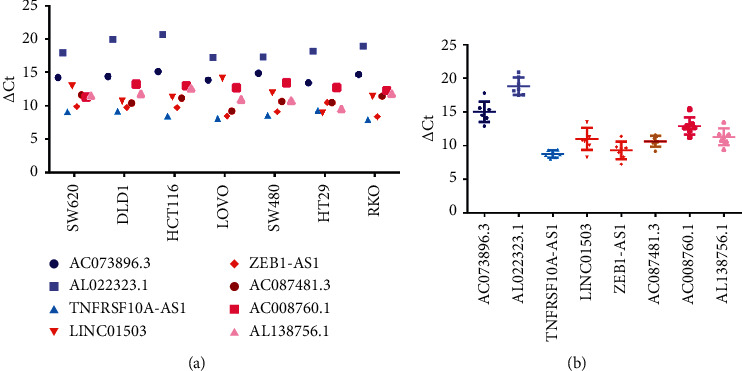
The expression levels of the eight autophagy-related lncRNAs in CRC cell lines. (a) The expression of the 8 lncRNAs in each cell lines. (b) The total expression levels of the eight autophagy-related lncRNAs in 7 cell lines. LncRNA, long noncoding RNA; CRC, colorectal cancer.

The Q-PCR assay was established to study the eight autophagy-related lncRNAs in cell lines of colorectal cancer (CRC). Seven cell lines of CRC were used in this study: SW620, DLD1, HCT116, LOVO, SW480, HT29, and RKO. The value of ΔCt was used to confirm the expression of the eight lncRNAs in cell lines. A higher value of ΔCt means a lower level of lncRNAs expression. The results indicated that five risk factors (LINC01503, ZEB1-AS1, AC087481.3, AC008760.1, and AL138756.1) were highly expressed in colorectal cancer, and two protective factors (AC073896.3 and AL022323.1) were low (Figure 8(a)).  Figure 8(b) shows the mean value of ΔCt of the each autophagy-related lncRNAs in all cell lines: AC073896.3 = 15.03, AL022323.1 = 18.8, TNFRSF10A-AS1 = 9.2, LINC01503 = 10.65, ZEB1-AS1 = 9.28, AC087481.3 = 10.31, AC008760.1 = 13.16, and AL138756.1 = 11.23. The results were generally consistent with our work.

## 4. Discussion

Colorectal cancer is a common type of malignant tumor. Owing to its rapid progression and easy metastasis, it has become the third leading cause of tumor-related mortality worldwide [18].

At present, colorectal cancer treatment involves surgery, chemotherapy, radiotherapy, biological therapy, etc. However, the effectiveness and prognosis are unsatisfactory. The 5-year survival rate of patients with colorectal cancer is only 42.7% [19]. Based on the widely utilized high-throughput biological technology, genomic alterations are accurately detected to predict the risk of metastasis and prognosis in patients with cancer [20, 21]. With the development of sequencing technology, investigators found that lncRNA plays an important role in transcription, posttranscriptional regulation, chromatin modification, splicing regulation, genomic imprinting, cell cycle regulation, and epigenetic regulation [22, 23]. This indicates that lncRNAs may be useful in predicting the prognosis of colorectal cancer. In previous studies, Zhou et al. found an effective lncRNA signature for predicting the risk of recurrence in colon tumors [24]. In addition, a risk model composed of mRNA and lncRNA could also be applied to detect the early recurrence of colon cancer [25]. These studies further prove the important role of lncRNA in tumors. Autophagy has been a hot topic in tumor research. However, there are few studies on the autophagy-related lncRNAs for patients with colorectal cancer. Therefore, it is necessary to construct the risk signature based on autophagy-related lncRNAs.

In the present study, the TCGA dataset was used to research the value of autophagy-related lncRNAs to predict prognosis of colorectal cancer. Firstly, we selected 860 autophagy-related lncRNAs through the co-expression of lncRNA and autophagy genes. Subsequently, eight autophagy-related lncRNAs were identified through multivariate Cox regression analysis. The risk signature was determined according to these autophagy-related lncRNAs, which can classify patients with colorectal cancer into high- and low-risk groups. Our results indicated that OS in the high-risk group was shorter than that noted in the low-risk group. Receiver operating curve analysis was used to validate the accuracy of the risk signature. The area under the curve of the risk score and stage was 0.689 and 0.695, respectively, indicating the risk signature's accuracy. Moreover, we further validated the risk model via univariate and multivariate Cox regression analyses. Moreover, we also performed Q-PCR assay to analyze the expression levels of the eight autophagy-related lncRNAs. Finally, we concluded that the risk signature based on these eight lncRNAs was an independent factor for colorectal cancer and was significantly associated with OS.

Autophagy is a highly conserved regulatory mechanism in eukaryotic cell evolution and an important degradation pathway for almost all cells from yeast to mammals [26]. More than 30 ATGs (e.g., ATG5, ATG12, and ATG16) are closely related to autophagy. The ATG12 binding process and LC3 modification process play important roles during autophagy. ATG12 is conjugated with ATG5 with the assistance of ATG7 to form the ATG5–ATG12 complex. LC3-I is finally transformed into LC3-II with the assistance of this complex, promoting autophagosome formation [27–29]. As mentioned above, autophagy plays different roles in tumors based on the type, stage, and genetic background of cancer [30]. It has been found that autophagy promotes the growth of cancer cells in a variety of tumor cells [31]. Under a stress state, the degree of autophagy increases to enhance cell adaptability, which is conducive to cell survival [32]. Yang et al. found that autophagy inhibition led to significant regression of tumors, which inversely proved that the degree of autophagy increased in pancreatic cancer cells [33]. In contrast to autophagy's potential role in promoting tumor proliferation, autophagy exerts an inhibitory effect on tumor growth. At present, the mechanism through which autophagy is induced to inhibit tumor development is not fully understood. Thorburn et al. found that autophagy prevented early steps in developing epithelial neoplasms [34]. It was also reported that autophagy could prevent tumorigenesis by limiting necrosis and chronic inflammation [35]. Our results showed the ATG12 was co-expressed with ZEB1-AS1, AC073896.3, AL138756.1, TNFRSF10A-AS1, and AC008760.1. In our risk model, AC073896.3 and TNFRSF10A-AS1 were protective factors, whereas ZEB1-AS1, AL138756.1, and AC008760.1 were risk factors. The results of this study indicated that the identified ATGs might exert different effects on colorectal cancer through different lncRNAs. Furthermore, this also reflected the complex functions of autophagy in tumors.

As described in Section 3, among the eight autophagy-related lncRNAs, three lncRNAs were protective factors (AC073896.3, AL022323.1, and TNFRSF10A-AS1) and the remaining five lncRNAs were risk factors (LINC01503, ZEB1-AS1, AC087481.3, AC008760.1, and AL138756.1). We also performed Q-PCR assay to explore the expression of these lncRNAs in cell lines. The expression of 7 lncRNAs was consistent with the previous results: 2 protective factors were low expression and five risk factors were high in CRCs. However, the protective factor, TNFRSF10A-AS1, is expressed highly in CRCs. We thought it might be due to the feedback upregulation during the development of the tumor. At present, only LINC01503 and ZEB1-AS1 have been studied extensively. Xie et al. found that the expression of LINC01503 was increased in squamous cell carcinoma, and its overexpression can contribute to the malignant phenotypes of squamous cell carcinoma cells in terms of cell proliferation, migration, and invasion [36]. Moreover, LINC01503 can promote proliferation and metastasis in gastric tumor cells through the WNT signaling pathway [37]. A previous study indicated that LINC01503 promoted the proliferation and metastasis of colorectal tumor cells by regulating miR-4492/FOXK1 signaling [38]. ZEB1-AS1 is one of the most characteristic oncogene regulatory factors in tumor-related lncRNAs and is often overexpressed in a variety of cancers [39]. Li et al. suggested that ZEB1-AS1 was upregulated in hepatocellular carcinoma (HCC) and promoted metastasis; hence, it can be used to predict the prognosis of this disease [40]. In addition to HCC, ZEB1-AS1 can also promote metastasis in numerous cancer types, such as prostate cancer, glioma, bladder cancer, and colorectal cancer [41–44]. In colorectal cancer, the OS and recurrence-free survival rates of patients with increased ZEB1-AS1 expression were lower, indicating that ZEB1-AS1 is a promising biomarker in predicting the clinical outcome [44]. In this study, LINC01503 and ZEB1-AS1 were associated with autophagy and may play a role in colorectal cancer prognosis. Moreover, the other autophagy-related lncRNAs we found may also help predict the prognosis of colorectal cancer, although most of them have been poorly studied.

Studies have shown that lncRNA is a novel regulator of autophagy. The lncRNA maternally expressed 3 (MEG3) is significantly downregulated in glioma tissues and cell lines, and its over expression can significantly inhibit cell proliferation and promote the apoptosis and autophagy of glioma cells [45]. The specific mechanism of lncRNA for the regulation of autophagy can be divided into three categories: (1) lncRNA acts as a competitive endogenous RNA-binding miRNA to regulate miRNA expression, thereby affecting the process of autophagy [46]; (2) lncRNA can also affect the expression of ATG genes and (3) lncRNA promotes tumor progression by regulating autophagy-mediated apoptosis through signaling pathways, such as the AKT/mTOR signaling pathway [47]. Recent studies revealed the relationship between signaling pathways and autophagy. The mTOR signaling pathway is crucial in regulating autophagy. When cellular nutrients and growth factors are abundant, mTOR1 is activated and phosphorylates key autophagy-related proteins, leading to the inhibition of autophagy; on the contrary, inhibition of mTOR1 leads to the induction of autophagy [48]. RAS signal transduction can also regulate autophagy through two main cellular pathways. Activation of RAS leads to increased stimulation of the PI3°K pathway and upregulation of mTOR1, resulting in inhibition of the autophagy system [49]. Furthermore, RAS activation can lead to increased autophagy by reducing the signal transduction of the mitogen-activated protein kinase/extracellular signal-regulated kinase (MAPK/ERK) pathway [49]. In colorectal cancer, studies found that autophagy was induced in HT-29 colon cancer cells following the stimulation of the MAPK/ERK signaling pathway [50]. Our results of the GSEA demonstrated that KRAS signaling gene sets were significantly enriched in the high-risk group, indicating that the selected eight autophagy-related lncRNAs were associated with KRAS signaling. This pathway may regulate autophagy, affecting the prognosis of patients with colorectal cancer.

## 5. Conclusion

We established a co-expression network of autophagy and related lncRNAs. Subsequently, the risk signature based on the eight autophagy-related lncRNAs was determined, and the risk model can predict the prognosis of patients with colorectal cancer. Owing to the various functions of lncRNAs, these autophagy-related lncRNAs may be promising targets for treating colorectal cancer. The results of this study provide new insight into exploring the prognosis of colorectal cancer.

## Figures and Tables

**Table 1:** Top 10 negative and positive co-expression of autophagy genes and related lncRNAs based on the value of the correlation coefficient.

ARGgene	lncRNA	Cor.	*p* value
*Negative-cor*			
MLST8	EBLN3P	−0.545241219	2.66*E* − 45
CAPN1	PSMA3-AS1	−0.532515097	6.47*E* − 43
CAPN1	EBLN3P	−0.529022775	2.81*E* − 42
MLST8	OIP5-AS1	−0.528187236	3.98*E* − 42
CAPN1	AC125257.1	−0.517555071	3.08*E* − 40
GABARAP	AC020663.2	−0.514900124	8.92*E* − 40
CAPN1	OIP5-AS1	−0.514192401	1.18*E* − 39
MLST8	PSMA3-AS1	−0.508377463	1.17*E* − 38
GAPDH	NORAD	−0.507132874	1.89*E* − 38
CAPNS1	AL049840.3	−0.506356429	2.56*E* − 38

*Positive-cor*			
BIRC6	Z68871.1	0.857358951	2.19*E* − 165
WDFY3	AL157392.3	0.845248067	3.62*E* − 156
WDFY3	AC097376.2	0.844822776	7.37*E* − 156
NAMPT	HIF1A-AS2	0.843125064	1.24*E* − 154
WDFY3	AC004492.1	0.841950039	8.55*E* − 154
WDFY3	SCARNA9	0.841546127	1.66*E* − 153
WDFY3	AC021078.1	0.840878641	4.92*E* − 153
WDFY3	USP12-AS1	0.840861569	5.05*E* − 153
PTEN	AL163051.2	0.837804156	6.92*E* − 151
WDFY3	SDCBP2-AS1	0.834270357	1.80*E* − 148

**Table 2:** The information of 32 selected lncRNAs significantly related to overall survival in colorectal cancer.

lncRNA	KM	B	SE	HR	HR.95 L	HR.95H	*p* value
LINC01503	0.044	0.233	0.076	1.262	1.087	1.466	2.25*E* − 03
AC026471.4	0.007	0.130	0.040	1.139	1.053	1.232	1.15*E* − 03
AC007128.1	0.035	0.316	0.160	1.372	1.003	1.876	4.79*E* − 02
AC011462.4	0.034	0.418	0.123	1.519	1.194	1.932	6.71*E* − 04
AL353194.1	0.038	0.148	0.075	1.159	1.000	1.343	5.00*E* − 02
AC099850.3	0.024	−0.062	0.025	0.940	0.895	0.986	1.17*E* − 02
AC004148.2	0.004	0.144	0.073	1.155	1.002	1.332	4.67*E* − 02
AC068580.3	0.002	0.336	0.125	1.400	1.096	1.788	7.11*E* − 03
ZEB1-AS1	0.001	0.766	0.172	2.150	1.534	3.014	8.74*E* − 06
AC087481.3	0.038	0.177	0.074	1.194	1.033	1.381	1.67*E* − 02
AL354836.1	0.005	0.114	0.038	1.121	1.041	1.206	2.39*E* − 03
AC005261.3	0.017	0.176	0.077	1.192	1.026	1.386	2.20*E* − 02
AC027796.4	0.021	0.381	0.092	1.464	1.222	1.753	3.42*E* − 05
AC083843.2	0.018	0.069	0.035	1.072	1.001	1.147	4.53*E* − 02
LINC02381	0.044	0.189	0.079	1.208	1.035	1.410	1.64*E* − 02
AC068580.1	0.033	0.261	0.104	1.298	1.058	1.593	1.23*E* − 02
LINC01011	0.030	0.350	0.144	1.420	1.070	1.883	1.51*E* − 02
AC010973.2	0.008	0.595	0.128	1.813	1.412	2.330	3.19*E* − 06
AC087741.1	0.029	0.421	0.143	1.524	1.152	2.015	3.13*E* − 03
AC107375.1	0.007	0.235	0.101	1.265	1.038	1.542	1.98*E* − 02
AC008760.1	0.032	0.450	0.119	1.568	1.243	1.980	1.52*E* − 04
AC073896.3	0.024	−0.517	0.221	0.596	0.387	0.919	1.91*E* − 02
SNHG7	0.012	0.035	0.014	1.036	1.007	1.066	1.38*E* − 02
AL138756.1	0.027	0.198	0.074	1.219	1.054	1.410	7.54*E* − 03
AL022323.1	0.004	−0.332	0.169	0.717	0.515	0.998	4.88*E* − 02
AL512413.1	0.010	0.349	0.128	1.418	1.102	1.824	6.57*E* − 03
AL161729.4	0.049	0.321	0.097	1.378	1.141	1.665	8.94*E* − 04
AL162586.1	0.000	0.402	0.107	1.495	1.211	1.845	1.79*E* − 04
TNFRSF10A-AS1	0.003	−0.138	0.057	0.871	0.780	0.973	1.43*E* − 02
LINC00174	0.013	0.249	0.095	1.283	1.065	1.544	8.62*E* − 03
AL022328.2	0.047	0.203	0.097	1.224	1.013	1.480	3.61*E* − 02
AC020558.2	0.013	0.378	0.159	1.459	1.068	1.991	1.75*E* − 02

**Table 3:** The coefficients and hazard ratio (HR) value of 8 autophagy-related lncRNAs estimated by multivariate Cox regression.

lncRNA	Coef.	HR
LINC01503	0.221971651	1.248535983
ZEB1-AS1	0.521879231	1.68519154
AC087481.3	0.200665779	1.222216213
AC008760.1	0.36500553	1.440521974
AC073896.3	−0.589465054	0.554623899
AL138756.1	0.161173845	1.1748892
AL022323.1	−0.35988948	0.697753438
TNFRSF10A-AS1	−0.141995146	0.867625468

**Table 4:** The association between the clinicopathological characteristics and risk score in colorectal cancer.

Clinical	Group	*n*	Mean	SD	*T*	*p*
Age	≤65	202	1.227	1.32	−0.946	0.345
	>65	271	1.336	1.119		

Gender	Female	222	1.159	0.912	−2.270	0.024
	Male	251	1.404	1.413		

Stage	Stage I-II	274	1.072	0.981	−4.434	0
	Stage III-IV	199	1.588	1.415		

T	T1-2	94	1.055	0.707	−2.961	0.003
	T3-4	379	1.347	1.298		

M	M0	373	1.182	1.096	−3.158	0.002
	M1	100	1.689	1.501		

N	N0	283	1.086	0.985	−4.241	0
	N1-2	190	1.592	1.432		

## Data Availability

The data used to support the findings of this study are included within the article.

## Abbreviations

lncRNA: Long noncoding RNA
TCGA: The Cancer Genome Atlas
ZEB1-AS1: ZEB1 antisense RNA 1
KRAS: Kirsten rat sarcoma viral oncogene homolog
mTOR: Mammalian target of rapamycin
COAD: Colon adenocarcinoma
READ: Rectum adenocarcinoma
ATG: Autophagy-related gene
GSEA: Gene set enrichment analysis
OS: Overall survival
CRC: Colorectal cancer.

## Supplementary Materials

Supplementary Materials Supplementary Materials. Figure S1: the prognostic effects of the previously published lncRNAs signature in colorectal cancer. (a) Survival analysis of the high- and low-risk groups according to the risk model in TCGA database. (b) The accuracy of the model was verified using ROC analysis. Table S1: the sequences of forward and reverse primers of the 8 autophagy lncRNAs.
