# Supplementary material for: An Autophagy-Related Long Noncoding RNA Signature Contributes to Poor Prognosis in Colorectal Cancer
Source: J Oncol. 2020 Oct 21;2020:4728947. doi: 10.1155/2020/4728947 (PMC7603611; doi:10.1155/2020/4728947)
Supplement: Supplementary Materials — Figure S1: the prognostic effects of the previously published lncRNAs signature in colorectal cancer. (a) Survival analysis of the high- and low-risk groups according to the risk model in TCGA database. (b) The accuracy of the model was verified using ROC analysis. Table S1: the sequences of forward and reverse primers of the 8 autophagy lncRNAs. [file 4728947.f1.docx]

**Supplementary Figure and Table:**

**
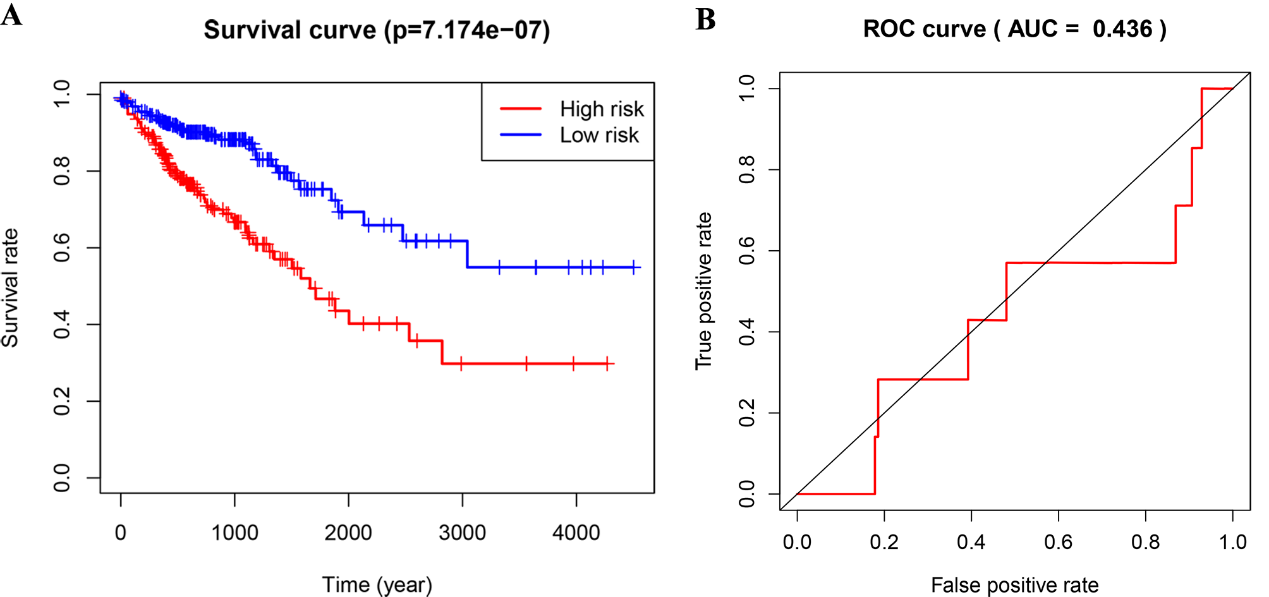
**

Fig S1: The prognostic effects of the previously published lncRNAs signature in colorectal cancer. (A) Survival analysis of the high- and low-risk groups according to the risk model in TCGA database. (B) The accuracy of the model was verified using ROC analysis.

**Table S1:** The sequences of forward and reverse primers of the 8 autophagy lncRNAs.

| LncRNA | Primers |
| --- | --- |
| AL022323.1-F | CTACGTGCCTCTTTCCCAGT |
| AL022323.1-R | AGCGTACTTCCTCCTTCCGA |
| AC087481.3-F | GACGTGCAAATGCCAGTGAA |
| AC087481.3-R | CTCCAGCGAGTGTCCAGTTT |
| AC073896.3-F | ACCTTTGAAATGTTTATTCAGGTCT |
| AC073896.3-R | AAGCACTCTGAGCTTGAGGT |
| TNFRSF10A-AS-F | TAGGATGAGAGCTGCCCACT |
| TNFRSF10A-AS-R | GGCCGTCCAGTAAGCTAAGG |
| AC008760.1-F | TTGCTCAGGGTTTCAGCGAT |
| AC008760.1-R | TGTCTATCCTGTCTCCCTTGTC |
| AL138756.1-F | GTCTTTTTCCTTATGTTGGACCTT |
| AL138756.1-R | TAGCCAACCATTTAGCAACGA |
| ZEB1-AS1-F | TCGCCTACCGAATCAGGTCA |
| ZEB1-AS1-R | CTTAGCAGGGAGGTACAGGC |
| LINC01503-F | CTGAATACACCCTCCCCACG |
| LINC01503-R | CTAGGGCTCTGCATTCGTCC |
